# Platelet detection as a new liquid biopsy tool for human cancers

**DOI:** 10.3389/fonc.2022.983724

**Published:** 2022-09-14

**Authors:** Maoshan Chen, Lijia Hou, Lanyue Hu, Chengning Tan, Xiaojie Wang, Peipei Bao, Qian Ran, Li Chen, Zhongjun Li

**Affiliations:** ^1^ Laboratory of Radiation Biology, Department of Blood Transfusion, Laboratory Medicine Centre, The Second Affiliated Hospital, Army Medical University, Chongqing, China; ^2^ State Key Laboratory of Trauma, Burns and Combined Injuries, The Second Affiliated Hospital, Army Medical University, Chongqing, China

**Keywords:** liquid biopsy, platelets, tumor-educated platelets, cancer, diagnosis, prognosis, biomarkers

## Abstract

Cancer is still a leading cause of death worldwide and liquid biopsy is a powerful tool that can be applied to different stages of cancer screening and treatment. However, as the second most abundant cell type in the bloodstream, platelets are isolated through well-established and fast methods in clinic but their value as a BioSource of cancer biomarkers is relatively recent. Many studies demonstrated the bidirectional interaction between cancer cells and platelets. Platelets transfer various proteins (e.g., growth factors, cytokine, chemokines) and RNAs (e.g., mRNA, lncRNA, miRNA, circRNA) into the tumor cells and microenvironment, leading the stimulation of tumor growth and metastasis. In turn, the platelet clinical characteristics (e.g., count and volume) and contents (e.g., RNA and protein) are altered by the interactions with cancer cells and this enables the early cancer detection using these features of platelets. In addition, platelet-derived microparticles also demonstrate the prediction power of being cancer biomarkers. In this review, we focus on the clinical applications of platelet detection using the platelet count, mean platelet volume, platelet RNA and protein profiles for human cancers and discuss the gap in bringing these implementations into the clinic.

## Introduction

As cancer is still a worldwide leading cause of morbidity and mortality, continuous efforts are made to improve the diagnosis and management of this disease. In recent years, precision medicine gained attentions in the tumor field as it aims to tailor therapies for patients in relation to the personalized patterns of the tumor (1–3). Early diagnosis and monitoring of disease progression are of great importance to improve the efficacy of therapies and to reduce cancer mortality (4). Although traditional tissue biopsy is the gold standard for tumor profiling, it presents some limitations (5), such as invasive and risky procedure, charged with potential complications, sometimes unrepeatable and could be unavailable. Furthermore, tissue biopsy provides a tumor picture limited to a single point at time and may show the genetic heterogeneity of various tumor subclones. Additionally, the dynamic genetic and epigenetic profiles of tumor cells further limit the tissue biopsy to capture the alternations of different tumor sites (4). Given this, the new field of oncology research has focused on the cancer-derived components that circulate in the bloodstream (6).

**Figure 1:**
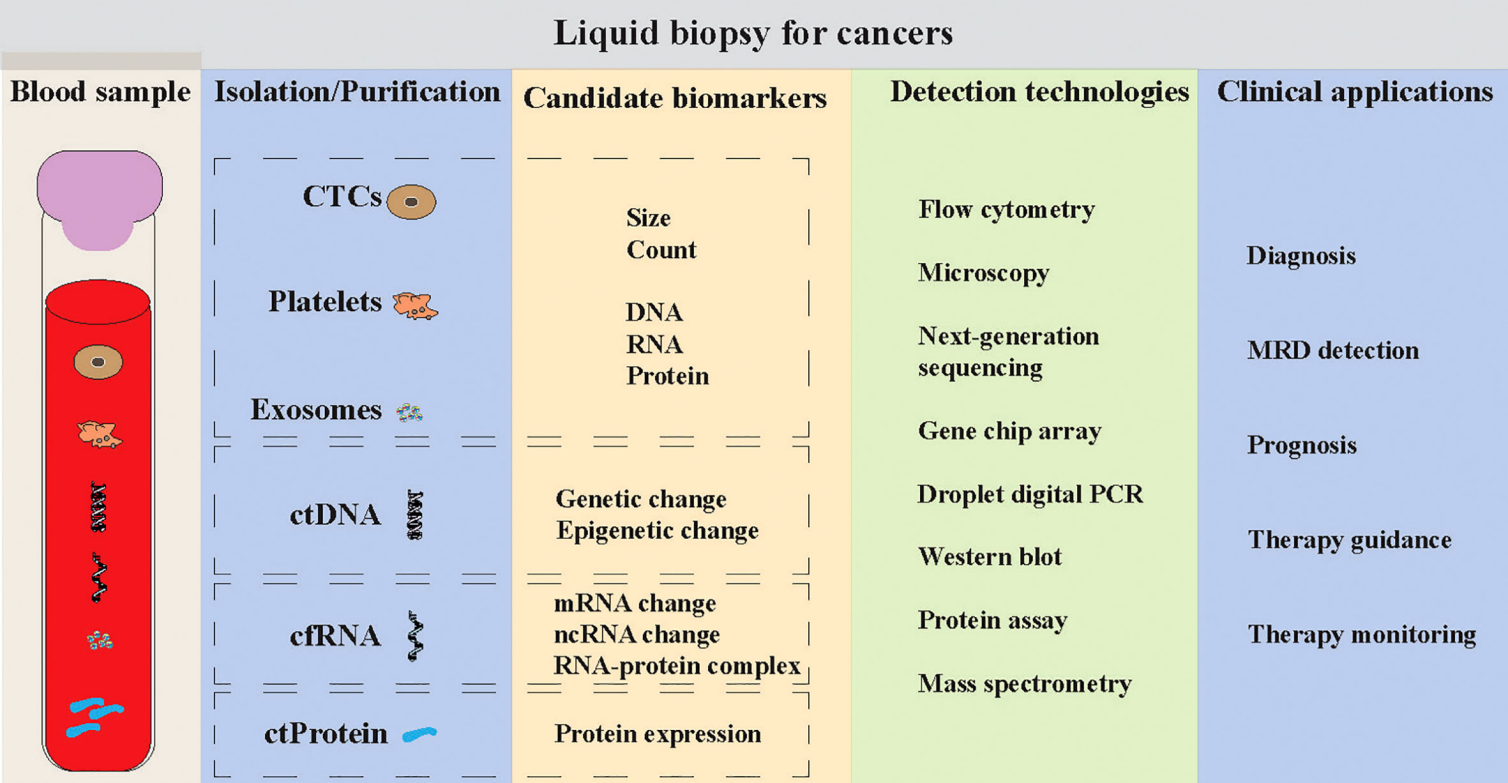
Liquid biopsy for human cancers. Liquid biopsy mainly consists of the detections of circulating tumor cells (CTCs), platelets, exosomes, circulating tumor DNA (ctDNA), circulating cell-free RNA (cf-RNA) and circulating tumor-associated protein (ctProtein). Multiple technologies can be applied to detect the tumor cell-associated information within these sources, such as morphological characteristics, DNA mutations, RNA expression, protein expression and epigenetic changes. The clinical applications of these liquid biopsy tools can be used for early cancer detection, minimal/measurable residual disease (MRD) detection, and monitoring the prognosis, therapy guidance and therapy monitoring of cancer patients.

Liquid biopsy is always introduced for the analysis of circulating tumor cells (CTCs), circulating tumor DNA/RNA/protein (ctDNA/ctRNA/ctProtein) and exosomes (**Figure 1**) (6–9). It may provide the opportunity of detecting, analyzing, and monitoring cancer in various body effluents such as blood or urine instead of a fragment of cancer tissue. It could give an accurate and comprehensive snapshot of the tumor and its microenvironment on multiple levels (10). Many studies have used the liquid biopsy approaches to improve the cancer screening, diagnosis and prognosis, the classification of more heterogeneous entities, the assessment of treatment response, and the detection of treatment-resistant subclones (4, 6), due to the advantages of liquid biopsy such as non-invasive, repeatable and real-time (3–5, 10). More recently, the blood platelets (thrombocytes), a neglected BioSource of tumor cell information, showed their vast potential in liquid biopsy (11, 12).

Platelets are small enucleated cellular fragments that are 2 – 4 µm in diameter and derived from megakaryocyte cells in the bone marrow. They are the second most numerous corpuscles in the blood normally circulating at between 150 – 450 × 10^9^/L (13). The lifespan of platelets in bloodstream is relatively short, ranging from 8 to 11 days, and subsequently degraded in the spleen (14). Since platelets were first described in 1881 (15), they have been shown to possess an important biological role at several stages of malignant disease (16), such as angiogenesis, cell proliferation, cell invasiveness and metastasis. The bidirectional interaction between cancer cells and platelets is evidently reciprocal and has been well reviewed by Yu et al. (17). Bioactive molecules on the membrane of and/or within the platelets are thought as key players in the cancer progression and metastasis (18). At the same time, the protein and RNA profiles of platelets can be altered by the interactions between cancer cells and platelets (16, 17). Thus, platelets are considered as important repositories of potential RNA (messenger RNA, microRNA, circular RNA, long noncoding RNA, and mitochondrial RNA) and protein biomarkers for early cancer detection, monitoring the disease progression and responses to treatment. Furthermore, several promising studies showed the abnormal clinical features (e.g., platelet count and mean platelet volume) of platelets from patients with cancer at early stages (19, 20). In the current paper, we will briefly review the crosstalk between cancer cells and platelets and present an overview of literature describing the clinical studies of platelet detection in patients with cancer in terms of platelet count, mean platelet volume, RNA/protein profile, and platelet derived microparticles.

## Crosstalk between cancer cells and platelets

**Figure 2:**
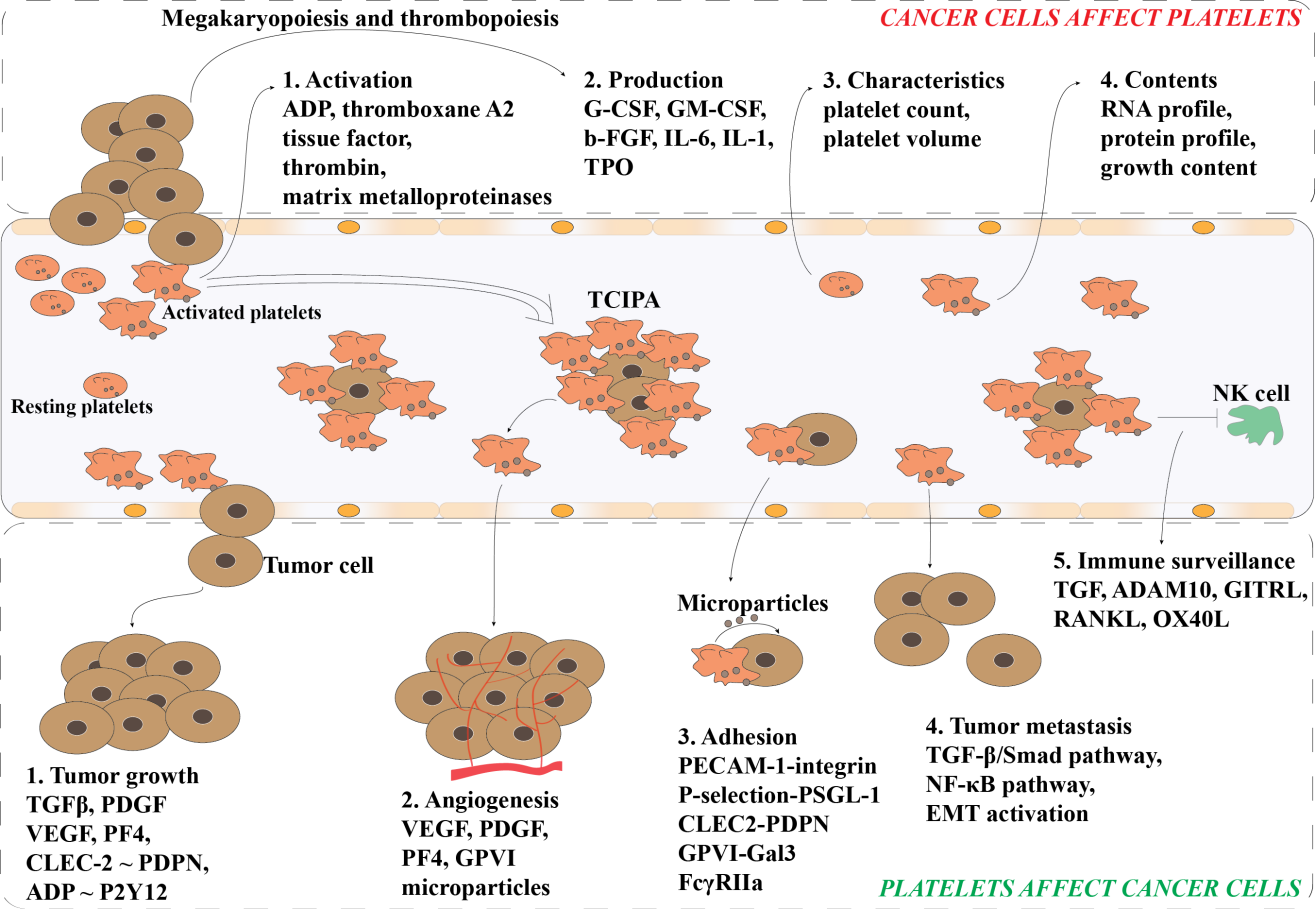
Bidirectional interactions of platelets and tumor cells. Cancer cells promote the platelet production and activation *via* the megakaryopoiesis and thrombopoiesis. Also, the morphological characteristics of platelets (e.g., count and volume) and contents within the platelets (e.g., RNA and protein) can be altered by cancer cells. Activated platelets can release various of mediators to facilitate the tumor growth and cancer metastasis. Activated platelets attach to the circulating cancer cells (CTCs) and enhance their survival in circulation *via* preventing CTCs from shear flow, immune surveillance, and apoptosis. Further, activated platelets also facilitate the adhesion and angiogenesis of CTCs. TCIPA, Tumor Cell-Induced Platelet Aggregation; G-CSF, Granulocyte- Colony-Stimulating Factor; GM-CSF, Granulocyte-Macrophage-Colony-Stimulating Factor; b-FGF, Basic Fibroblast Growth Factor; IL-1, Interleukin-1; IL-6, Interleukin-6; TPO, thrombopoietin; TGFβ, Transforming Growth Factor β; PDGF, Platelet-Derived Growth Factor; VEGF, Vascular Endothelial Growth Factor; PF4, Platelet Factor 4; PDPN, Podoplanin; CLEC2, C-type lectin receptor type 2; ADP, Adenosine 5’Diphosphate; P2Y12, platelet P2Y12 receptor (P2Y12R) for ADP; GPVI, Platelet Glycoprotein VI; PECAM-1, Platelet-Endothelial Cell Adhesion Molecule-1; PSGL-1, P-selectin Glycoprotein Ligand-1; Gal3, Galectin-3; FcγRIIa, The Platelet Fc Gamma Receptor II Type a.

Although platelets are produced by the megakaryocytes in the bone marrow sinusoids, the crosstalk between cancer cells and platelets happens in multiple ways and all mechanisms inducing platelets activation and aggregation are called tumor cell induced platelet aggregation (TCIPA) (21). Clinical data have shown that cancer patients often suffer from thrombotic complications (e.g., deep vein or arterial thrombosis, and pulmonary emboli) and that thrombosis is the second leading cause of malignancy-associated death (22, 23). The impacts of cancer on platelets reviewed by Plantureux et al. showed that cancer cell-induced platelet production, activation and function alteration might be the major reasons of thrombosis (**Figure 2**) (21). They summarized that a variety of tumor-related humoral factors and cytokines directly or indirectly influence megakaryopoiesis and thrombopoiesis during cancer progression, such as granulocyte colony-stimulating factor (G-CSF), granulocyte-macrophage colony-stimulating factors (GM-CSF), basic fibroblast growth factor (b-FGF), interleukin-6 (IL-6), interleukin-1 (IL-1), and thrombopoietin (TPO). Yu *et al.* reviewed that cancer cells activate platelets by direct cell interactions or by releasing various mediators, such as ADP, thromboxane A2, tissue factor, thrombin and matrix metalloproteinases (17). Additionally, the growth factor contents, RNA and protein profiles of platelets, and platelet characteristics (e.g., platelet count and volume) are also influenced by cancer cells (17). The alternation of platelet characteristics, mRNA and protein content makes platelets an interesting new target for blood biomarker research for the detection of early-stage cancer, which will be discussed later in this study.

In turn, platelets can stimulate angiogenesis and induce cancer growth, proliferation, and metastasis (**Figure 2**). Platelets contain various bioactive growth factors including chemokines, cytokines, and matrix metalloproteinases (MMPs) and their secretion into the tumor microenvironment support platelets playing an important role in the regulation of tumor angiogenesis, vascular stabilization and integrity, and resistance to therapy (16). Among the bioactive growth factors, transforming growth factor β (TFGβ) (24, 25), platelet-derived growth factor (PDGF) (25), and vascular endothelial growth factor (VEGF) (26, 27) are three well known growth factors released by platelets that can promote the proliferation of cancer cells. In addition, platelet associated platelet factor 4 (PF4) and interactions of CLEC-2~podoplanin and ADP~P2Y12 were found to expedite the tumor growth in several cancer types including lung (28, 29) and ovarian (30). Additionally, the secretome and microparticles released by activated platelets also increase the proliferation of some cancer cells, such as ovarian and hepatocellular carcinoma cancer cells (24, 31). The formation of circulating tumor cell (CTC)-platelet aggregates enables the protection of CTCs from immune surveillance and the adhesion of platelets to the endothelium of potential metastatic sites (32, 33). Furthermore, the interaction of platelet and tumor cells can activate the TGF-β/Smad and NF-κB pathways in malignant cells, and further enhance the aggressiveness of tumor cells *via* epithelial-mesenchymal transition (EMT) (33–35). The expression of some mesenchymal markers, such as SNAIL, vimentin, fibronectin, MMP-9, and E-cadherin, in the cancer cells can be modulate by platelets (34, 36).

## Clinical applications of platelets for cancer patients

### Platelets counts

Thrombocytosis has been well known as an indicator of health outcomes not necessarily confined to thrombosis and haemostasis processes (37). In 2000, based on the platelets of stage IV renal cell carcinoma (RCC) patients who had undergone a variety of adjuvant therapies after nephrectomy, Symbas *et al.* first reported that thrombocytosis (platelet count > 400,000/μL) was a negative predictor for survival in metastatic RCC (38). However, this was not consistent with the study performed in 2001 by Négrier et al. (39). This issue was then addressed by Petros’s group themselves in another study cohort of 204 patients with RCC who underwent radical nephrectomy with curative intent between June 1993 and January 2000 at Emory University Hospital (40). They again documented the association of thrombocytosis with decreased survival of RCC patients and found that the cancer specific death rate of early stage RCC patients who undergo nephrectomy with a perioperative platelet count of greater than 400,000/μL was greater than 5 times the rate in patients with a persistently normal platelet count after radical nephrectomy. In 2006, another cohort study of 700 previously untreated metastatic RCC patients enrolled on phase 1, 2, or 3 clinical trials conducted in the United States and Europe confirmed thrombocytosis as an independent prognostic factor for survival in patients with metastatic RCC (41). Further, the platelet count of RCC patients was found with strong correlation with T stage, tumor size, Fuhrman grade, nodal invasion, hemoglobin level, lymph node positivity, ECOG score, and the presence of distant metastasis (42, 43). The 5-year survival rate was 70% for the patients with a platelet count < 450,000/μL, compared to 38% for the patients with the platelet count ≥450,000/μL (42).

In other solid tumors, elevated platelet count is also associated with poor prognostics. Long *et al.* performed a meta-analysis of thirty colorectal cancer (CRC) studies and reported that pre-treatment elevated platelet count was associated with poorer overall survival (Hazard ratio = 1.837, 95% confidence interval, 1.497 to 2.255, p = 0.000) and poorer disease-free survival (Hazard ratio = 1.635, 95% confidence interval, 1.237 to 2.160, p = 0.001) in CRC patients (44). In ovarian cancer, the decrease of platelet count can significant prolong overall survival and progression-free survival in serous ovarian cancer patients (45). The overall hazard ratio, progression-free survival and disease-free survival were 1.81 (95% CI: 1.52 – 2.15), 1.48 (95% CI: 1.24 – 1.75) and 1.39 (95% CI: 1.19 – 1.61), respectively, in ovarian cancer patients with elevated pre-treatment platelet count (46). In a large cohort study of Chinese Taipei population, both low and high platelet count in older cancer patients was observed to associate with high mortality, presenting a U-shaped curve, which was further confirmed by a similar cohort study in Danish population (47, 48). Although some studies showed that thrombocytopenia is a poor prognostic factor for cancer patients (49, 50), a nested case-control study over a 10-year period gave a final answer in 2022 that an elevated platelet count was associated with cancer at several sites and could potentially serve as a marker for the presence of some cancer types (51).

### Mean platelet volume

Mean platelet volume (MPV) is the average size of platelets, which is measured by hematological analyzers based on the volume distribution during routine blood morphology test. The normal MPV is about 9.4 to 12.3 fL, whereas the percentage of large platelets amounts to 0.2 ~ 5.0% of the whole platelet population. Probably due to the haemostasis maintenance and preservation of constant platelet mass, MPV is inversely proportional to the platelets (52). Considering increased platelet is associated with poor prognostics of cancer patients, theoretically MPV could be another potential marker for human cancers. However, the actual situation might be different. In a Danish population cohort study, adjustment for MPV did not change the association between platelet count and mortality of cancer patients, although MPV correlated inversely with the platelet count (Spearman’s rho = -0.41, p < 0.001) (48). Some studies demonstrated that increased MPV is a marker for cancers. By comparing the MPV levels of 230 subjects with normal, chronic hepatitis, cirrhosis, and hepatocellular carcinoma (HCC), Kurt *et al.* showed that MPV might be a potential or adjunctive marker for HCC patients with chronic liver diseases (53). Another study by Cho *et al.* also showed the higher levels of MPV and high ratio of MPV/platelet count in HCC patients compared to healthy individuals (54). The increase of MPV as a marker has also been observed in patients with CRC (55), metastatic CRC (56), gastric cancer (57), papillary thyroid cancer (58, 59), pancreatic cancer (60), and oral cancer (61). Interestingly, no change of or decreased MPV was also observed between cancer patients and healthy subjects in the studies of CRC (62), lung cancer (63, 64), uterine cervix (65), RCC (66), oral cancer (67), and prostate cancer (68).

Although researchers have divergent findings of the MPV levels in cancer patients and healthy control, it is agreed by most studies that decreased MPV is a good prognostic biomarker for cancer patients. In CRC patients, decreased MPV was observed after surgical removal of intestinal tumor (55) or chemotherapy (69). Further, CRC patients with lower MPV were found to respond much better to the chemotherapy and achieve longer remission (56). High MPV increased the fatality by 4.7 times (p = 0.032) for oral cancer patients (61). The decrease of MPV when treatment applied was also reported in the patients with pancreatic cancer (60, 70), squamous cell carcinoma of the esophagus (71), and gastric cancer (72). Compared with other clinical covariates (e.g., neutrophil-to-lymphocyte ratio, platelet-to-lymphocyte ratio, combined neutrophil-platelet score and systemic immune-inflammation index), the ratio of MPV/platelet count was more accurate in determining the prognosis of gastric cancer patients and could be an independent prognostic factor for patients with resectable gastric cancer (73). Further, some studies investigated the cause of increased MPV in cancer patients. In gastric cancer, the increase of MPV was associated with chronic inflammation and elevated IL-6 concentration, which causes the maturation and proliferation of megakaryocyte and the enhanced release of platelets (74). The increase of IL-6 was observed in the serum of gastric cancer patients by different groups (74–76). Another cause might be the genetic variants, which have influence on the biogenesis and reactivity of platelet (77–79), such as megakaryocytopoiesis, megakaryocyte/platelet adhesion, platelet formation, platelet count and MPV. However, the molecular mechanisms and regulation networks of IL-6 and other mutated genes in the platelet count and MPV remain not fully understood and require more future research.

### Tumor-educated platelets contain clinical RNA biomarkers

As an important part of the tumor microenvironment, the alternation of platelet RNA profiles *via* direct and indirect communications with tumor cells or tumor cell derived biomolecules is called education of platelets by tumor cells, and the platelets after this process are so called tumor-educated platelets (TEP). Although the term of “TEP” was first described by Best *et al.* in 2015, the observation of tumor affecting platelets can date back to 19th century. In 1865, Trousseau observed that cancer cells can induce the thrombosis formation (80), which is called “Trousseau’s syndrome” or cancer-associated thrombosis (81). The direct interaction between tumor cells and platelets was observed by Billroth in 1877 (82), and they found the “thrombi filled with specific tumor elements” as part of tumor metastasis (83, 84). As more advanced technologies were applied into the identification of biomolecules within platelets, studies have been demonstrated to search tumor-derived RNA biomarkers in platelets. Using microarray analysis, Gnatenko *et al.* identified approximately 2,000 transcripts (13 ~ 17% of probed genes) in unstimulated platelets and concluded that the platelet transcriptome will be another option to study proteins related to normal and pathologic platelet functions (85). In another gene microarray study performed by Calverley in 2010, 197 out of 200 platelet genes were downregulated in lung cancer metastasis and 33 of them were identified with between 3 and 13 splicing events each (86). In 2011, Nilsson *et al.* reported that cancer-associated RNA biomarkers EGFRvIII and PCA3 were transferred into the platelets isolated from glioma and prostate cancer patients, respectively (87). They also performed the gene expression microarray and identified distinct RNA signatures in platelets from glioma patients compared with normal control subjects. They concluded that platelet transferring of tumor RNA molecules is independent from the microvesicle transfer mechanisms in the circulating system (87).

As deep sequencing technology and computer identification algorithms developed in the past few years, they have been applied into the identification of clinical biomarkers in the platelets isolated from cancer patients. Best *et al.* applied SMARTer mRNA amplification for the low amount (100 ~ 500 pg) of total RNA and performed RNA sequencing for TEP of multiple cancer types, including non-small cell lung cancer (NSCLC), CRC, glioblastoma (GBM), pancreatic cancer (PAAD), hepatobiliary cancer (HBC) and breast cancer (BrCa) (88). They identified platelet marker genes (e.g., B2M, PPBP, TMSB4X and PF4) in the TEPs and found that the platelet RNA profiles were correlated with previously reported platelet and megakaryocyte but not with other blood cell RNA profiles. Using a leave-one-out cross-validation support vector machine algorithm (SVM/LOOCV), 1,072 platelet RNAs were selected to build a predictive model for pan-cancer, which gave an accuracy of 95% in the training cohort and 96% in the validation cohort (88). Then, the authors applied this method into the prediction of multiclass cancer diagnostics and obtained 84% ~ 100% accuracy in the validation cohorts of individual cancer subtype. When the TEP RNA profiles were used to discriminate specific cancer in three (CRC, PAAD and HBC) and all types of adenocarcinomas, the overall accuracy of prediction classifiers in validation cohorts was 76% and 71%, respectively. Two years later in 2017, Best *et al.* showed high accuracy of detecting early- (81%) and late-stage (88%) of NSCLC using TEP transcriptome profiles, independent of age, smoking habit, whole-blood storage time and various inflammatory conditions (89). In a liquid biopsy study of endometrial cancer, the accuracies of utilizing TEP transcriptome profiles and circulating tumor DNA (ctDNA) coupled with artificial intelligence were compared and the authors reported higher accuracy and AUC of TEP-dedicated classifier in predicting endometrial cancer (90). Xu *et al.* reported that TEP RNA profiles have high potential of distinguishing and staging CRC patients from other noncancerous intestinal diseases (91). When Xiao *et al.* used the pan-cancer model from Wurdinger (88) to predict RCC using the TEP RNA profiles, it showed 48.7% accuracy and 0.615 AUC value (92). Their results showed that the pan-cancer model developed by *Best et al.* in 2015 may not be suitable for other cancer types and that more TEP RNA profiles need to be analyzed to improve the pan-cancer model.

In addition to the TEP mRNA profiles, some studies uncovered the noncoding RNA biomarkers within the platelets from cancer patients. Ye *et al.* reported four lncRNAs (e.g., LNCAROD, SNHG20, LINC00534 and TSPOAP-AS1) were upregulated in the platelets of CRC patients and a binary logistic model derived from these four lncRNAs can predict CRC with an AUC of 0.78 (93). Mantini *et al.* reported 41 and 981 differentially expressed miRNAs and isomiRs (miRNA isoforms produced by Drosha and Dicer), respectively, in pancreatic ductal adenocarcinoma (94). Further, platelet-derived circNRIP1, a circular RNA derived from the NRIP1 gene, was found as a potential diagnostic biomarker for NSCLC patients (95). These studies strongly support platelet detection as an alternative liquid biopsy tool for human cancers.

### Platelet derived proteins as cancer biomarkers

Upon the activation, platelets can transport numerous of bioactive proteins into the microenvironment, such as growth factors, chemokines and proteases (96). *In vivo* studies of tumor-bearing mice showed increased protein levels of tumor derived factors in platelets, such as TGF-β, MCP-1, RANK, TIMP-1, and TSP-1 (97, 98). The expression level of TSP-1 was highly correlated with tumor progression and was decreased after the tumor resection (98). Another *in vivo* study of mice bearing human tumors identified PF-4 upregulated in early growth of human liposarcoma, mammary adenocarcinoma and osteosarcoma (99). Thus, the upregulation of TSP-1 and PF-4 inside platelets was reviewed to detect clinically undetectable tumors (< 1 mm^3^) in mice (98, 99).

Studies have also been conducted to report potential protein markers for cancer patients since platelet proteome was remarkably altered in cancer patients (17). Sabrkhany *et al.* identified 85 proteins in platelets significantly changed in patients with early-stage lung and pancreas cancers compared to controls (100). It is interesting that 81 of these 85 proteins restored their expression to normal level after the tumor resection. Peterson *et al.* demonstrated elevated concentrations of VEGF, PF-4 and PDGF in the platelets of CRC patients (101). These three platelet proteins were analyzed to be independent predictors of CRC and as a set providing a significant discrimination (AUC: 0.893, P < 0.001). Sabrkhany *et al.* demonstrated a significant increase of VEGF and PDGF concentrations in the platelets of patients with early-stage (I-II) lung cancer compared to the platelets of a healthy sex- and age-matched control group (20). However, in the platelets of late-stage (III-IV) lung cancer patients PF-4, CTAPIII, and TSP-1 were decreased as compared to the control group. In patients with head of pancreas cancer only, VEGF was elevated in platelets as compared to controls (20). This study also showed that the utilization of a combination of platelet features (including clinical characteristics and protein contents) may improve the prediction power of discriminating cancer patients and healthy controls. Some platelet protein biomarkers were identified to discriminate benign adnexal lesions and ovarian cancer (FIGO stages III-IV) with high sensitivity and specificity (102). Overall, these studies support that platelet derived proteins can also be utilized as biomarkers for cancers, however, how tumor cells modify platelet proteome remains unclear so far.

## Platelet-derived extracellular vesicles

EVs are nano-membrane particles (30 – 2000nm) released by most cell types and function in cell-cell communications (103, 104). Platelets transfer their contents by releasing two types of EVs – exosomes (40 – 100 nm) and microparticles (100 – 1000 nm), of which platelet-derived microparticles (PMPs) present the most abundant population of EVs in blood (105). It is well documented that PMPs are associated with many aspects of cancer progression, including angiogenesis, tumor growth, metastasis, escape from apoptosis and immune surveillance, extracellular matrix degradation, and chemoresistance (106, 107). To our knowledge very limited studies focused on the diagnostic applications of PMPs or platelet exosomes in cancer patients. However, D’Ambrosi *et al.* hypothesized that PMPs can be potentially used as a cancer biomarker, as an increase in the number of circulating EVs are observed in many types of cancer and the concentration of circulating PMPs increases with cancer development (107).

## Advantages and limitations of platelet detection

Platelet detection is an easy-to-go liquid biopsy method for human cancers. The preparation strategy of platelets does not require advanced instruments or commercial reagents. Best *et al.* introduced the thromboSeq pipeline that includes a platelet-isolation protocol with differential centrifugation steps and that can result in relatively pure platelet preparations (1 – 5 leukocytes per 1 million platelets) (108). As shown in **Figure 1**, like CTCs and exosomes platelets can be used to detect both clinical characteristics (e.g., size and count) and molecular biomarkers (e.g., RNA, and protein) for cancer patients. As infections (caused by bacteria and viruses), autoimmune diseases (e.g., lupus, immune thrombocytopenia, and idiopathic thrombocytopenic purpura), drugs (e.g., antibiotics that contain sulfa, heparin to prevent blood clots, and anti-seizure drugs such as phenytoin and vancomycin), and hemolytic uremic syndrome can also alter the count, mean volume and protein profiles of platelets in the patient blood, it is challenging to use these markers to detect cancers. Thus, platelet count, mean volume and some proteins might be only used as one of the symptoms for cancer detection currently. We summarized the advantages and limitations of different liquid biopsy methods in clinical applications (**Table 1**). Like platelet count and mean volume, the circulating tumor-associated markers like AFP, CEA, PSA, CA19-9 and CA72-4 might be limited to the aid diagnosis currently, because they are not sensitive or specific and not recommended for population screening (109). Although platelet detection for cancer is still in early development, the applications of TEP RNA profiles have demonstrated high sensitivity and accuracy in predicting human cancers and can be applied for early cancer diagnosis (86–95). Further, the purity of platelets and the cost for low-input deep sequencing are challenges that hinders the clinical use of TEP RNA profiles for cancer detection.

**Table 1:** Comparison of liquid biopsy methods for cancer diagnosis and prognosis.

Biomarker	Advantages	Limitations
Cirulating tumor cells (CTCs)	Broad utility; Photography of spatial and temporal tumor heterogeneity; Analysis of RNA expression (e.g., miRNA) and proteomics; Analysis of epigenetic alternations (e.g., methylation); Cell morphology and functional studies ex vivo;	Rare in circulation; Difficult and costly isolation;
Circulating tumor DNA (ctDNA)	Well-developed analysis techniques; Biobanking preservation; Analysis of DNA mutations; Analysis of epigenetic alternations (e.g., methylation); High specificity and accuracy;	Low concentrations and low sensitivity of detection; Rapidly degraded in plasma with short half-life; Lack of standardized preanalytical protocols;
Cell-free RNA (cfRNA)	Analysis of RNA expression and mutations; Stable in blood (e.g., miRNA);	Rapidly degraded in plasma with short half-life;Techniques in early development;
Exosomes	Can be found in blood, ascites, and pleural fluid; Biobanking preservation; Analysis of RNA expression and proteomics; Analysis of epigenetic alternations (e.g., methylation);	Lack of standardized preanalytical protocols;
Platelets	Highly stable in blood; Abundant; Potential use for multiple cancer–type screening; Analysis of RNA expression and proteomics; High specificity and accuracy;	Techniques in early development;
Circulating tumor proteins (e.g., AFP, CEA, PSA, CA19-9 and CA72-4)	Easy isolation; Fast test and low cost; Clinically used in aid diagnosis and prognosis;	Low sensitivity; Low specificity; Not suitable for population screening;

## Conclusions and perspectives

Liquid biopsy is an important tool for early cancer detection and allows the improvement of overall survival of cancer patients. Platelet detection is increasingly evident to be a useful tool of liquid biopsy for patients with cancer, like other liquid biopsy tools (e.g., CTC, ctDNA, cfRNA, exosomes) (**Figure 1**). This review summarizes and discusses the platelet features (e.g., count, volume, RNA profile and protein profile) and the clinical studies utilizing these features in the detection of cancers at different stages. It is believed that clinical characteristics of platelets (from normal blood test) including platelet count and mean platelet volume are altered in patients with cancer, however, they seem to have more prognostic value and their potential of being diagnostic biomarkers for early cancer detection require more studies to be validated. Further, the protein and RNA profiles of platelets showed high accuracy in the prediction of early cancer diagnosis and decision of tumor stages for patients. Remarkably, utilization of TEP RNA profile and artificial intelligence enables high specificity and sensitivity in the detection of several cancer types, but the model generated from one cancer cohort may not be applicable to another one. To our knowledge, the prediction tools of TEP RNA profiles have much higher accuracies in both train and validation datasets than other liquid biopsy tools. But they still require long-time follow up studies to improve the prediction power in early cancer diagnosis.

The combination of platelet features would improve the diagnostic model for discriminating cancer patients from healthy controls (20). This might enlighten us that the combination of platelet detection and other liquid biopsy tools might be future research directions of early cancer diagnosis. In addition, the detection technologies applied with platelet contents might limit the use of platelets in the diagnosis and prognosis for cancer patients. Although there are some problems that to be addressed for the clinical use of platelet detection for human cancers, such as the biomarker candidates, computing algorithms and detection technologies, platelets may become the holy grail in cancer blood biomarker research and will gain more attention from the industry.

## Author contributions

MC, LC and ZL conceived the project. MC and LHo prepared the initial draft. MC and LHo prepared the figures. LHu, CT, XW, PB, QR, LC and ZL contributed to the editing of the initial draft. All authors contributed to the article and approved the submitted version.

## Funding

This work was financially supported by the National Natural Science Foundation of China (81770197 and 81903838), the Young Talents Program of Chongqing (T03010008), and the Natural Science Foundation of Chongqing (cstc2020jcyj-msxmX0051).

## Conflict of interest

The authors declare that the research was conducted in the absence of any commercial or financial relationships that could be construed as a potential conflict of interest.

## Publisher’s note

All claims expressed in this article are solely those of the authors and do not necessarily represent those of their affiliated organizations, or those of the publisher, the editors and the reviewers. Any product that may be evaluated in this article, or claim that may be made by its manufacturer, is not guaranteed or endorsed by the publisher.
